# Metabolomic Insights into Sexual Multi-Morphism of Sinomenine Accumulation in *Sinomenium acutum*

**DOI:** 10.3390/plants14121885

**Published:** 2025-06-19

**Authors:** Yanxian Luo, Wen Xu, Yanling Fan, Xinyu Ma, Qian Deng, Meng Li, Wei Sun

**Affiliations:** 1 College of Life Science and Technology, Central South University of Forestry and Technology, Changsha 410004, China; 15206031383@163.com (Y.L.); fan_yanling@126.com (Y.F.); maxinyu302@163.com (X.M.); 20231200176@csuft.edu.cn (Q.D.); 2 Ministry of Agriculture and Rural Affairs of the Huaihua, Huaihua 418000, China; xuwenno.1@163.com; 3 Yuelu Mountain Laboratory, Carbon Sink Forest Variety Creation Center, Changsha 410004, China

**Keywords:** *Sinomenium acutum*, sinomenine, gender difference, metabolomics, abscisic acid (ABA), isoquinoline alkaloids, coclaurine, phytohormone

## Abstract

*Sinomenium acutum* is the main raw material for sinomenine. Empirical evidence indicates a marked disparity in sinomenine content among *S. acutum* plants with different genders, resulting in varying medicinal potential of the processing products. However, the mechanism underlying gender-determined differences in sinomenine accumulation is still elusive. In this study, untargeted metabolomics was performed among female, male, and undifferentiated *S. acutum* plants. In total, 1213 metabolites were identified, and most of them vary in the roots but not in the leaves among the different genders. Integrated correlation analysis on the DAMs (differentially accumulated metabolites) enriched in the isoquinoline alkaloid biosynthesis pathway suggests coclaurine as an intermediate determining gender-dependent sinomenine variation. Furthermore, hormonal profiling revealed 34 endogenous phytohormones exhibiting significant gender-based discrepancy in the roots. Among these, ABA (abscisic acid) and 5-DS (5-deoxystrigol) show significant positive correlation with sinomenine content. Then, exogenous ABA with gradient concentration was applied on *S. acutum* plants, and the sinomenine content in the roots increased from 31% to 166% under treatment. Our findings demonstrate that coclaurine might serve as a pivotal intermediate during sinomenine biosynthesis in *S. acutum*. Meanwhile, it is speculated that ABA is a key factor regulating different sinomenine accumulation, which provide a potential method to improve the yield of sinomenine.

## 1. Introduction

*Sinomenium acutum* is a perennial vine belonging to the Menispermaceae family within the Ranunculales order. It is a national protected variety of China and serves as an important raw material in traditional Chinese medicine. *S. acutum* was first documented for its medicinal value in the ancient Chinese book “Illustrated Materia Medica” from the Song Dynasty, and has a long history of being used to treat rheumatic diseases. According to the 2020 edition of the Pharmacopoeia of the People’s Republic of China the original plants of *S. acutum* include *Sinomenium acutum* (Thunb.) Rehd. et Wils and *Sinomenium acutum* (Thunb.) Rehd. et Wils. *var. cinereum* Rehd. et Wils, whose roots and stems are harvested in autumn or winter and then dried as a raw material of traditional Chinese medicine [1]. The wild *S. acutum* resources are primarily found in the central and southern regions of China, including areas such as Hunan, Hubei, and Guangdong Provinces. In addition, they are also distributed in other Asian countries such as Japan and Malaysia. The naturally distributed *S. acutum* plant usually grows at the edge of forests on hillsides, along ditches or in shrublands. It prefers a climate with abundant rainfall, cool, and moist conditions, and is suitable for growing in loose, fertile, and well-drained soil. For instance, there is abundant precipitation in the southern region of Japan, which is suitable for the growth of *S. acutum*. Japanese scholars isolated sinomenine from *S. acutum* plants as early as the 1950s [2].

The chemical composition of *S. acutum* includes quinone, anthraquinone, phenols, triterpenoids, volatile oils, and alkaloids, which are the primary medicinal components of this plant [3]. The alkaloids of *S. acutum* are composed of sinomenine, magnolamine, linaloolamine, acutamine, norlinaloolamine, palmatine, magnolamine, africanine, jatrorrhizine, and 8-hydroxypalmatine [4]. Among these, sinomenine is the most important ingredient. The chemical structure of sinomenine is similar to morphine with a physical form of long filamentous crystal [5,6]. Sinomenine and its hydrochloride form have significant therapeutic effects on rheumatoid arthritis [7,8], tumors [9,10], kidney damage [11], and liver diseases [12]. Currently, the raw materials of *S. acutum* are still manually collected from the wild, which causes resource damage, and the yield and quality cannot be guaranteed, resulting in inconsistent product quality. However, the factors determining sinomenine yield remain unclear and the quality (medicinal potential) of raw materials is still judged by experience. Therefore, deciphering the regulatory mechanism underlying sinomenine biosynthesis is crucial for the efficient utilization of *S. acutum* resources.

Sinomenine belongs to the isoquinoline alkaloids (IQAs). IQAs are a type of plant secondary metabolite with wide sources and various pharmacological activities. They are generally found in plants belonging to closely related families such as the Papaveraceae, Ranunculaceae, Annonaceae, and Chloranthaceae [13,14,15]. Most studies concerning sinomenine are focused on the biological activity of benzyl IQAs [16]. Extensive literature has verified that *L*-tyrosine is a common precursor in the IQAs biosynthesis pathway. *L*-tyrosine can be converted into a skeleton structure by polymerization with phenolic precursors and then modified by different enzymes to form IQAs. With the deepening of understanding of the IQAs biosynthetic pathway, the key enzymes and related genes were identified. Now, it has become possible to use a synthetic biology strategy to produce IQAs or enhance their production capacity [17]. The initiation steps of the biosynthetic pathway for various IQAs are similar. Most IQAs are synthesized from tyrosine to form dopamine. Under the action of NCS (Norcocalurine synthase), dopamine and 4-HPAA (4-hydroxyphenyl acetaldehyde) form (*S*)-norcoclaurine through the Pictet–Spengler reaction. This is followed by 6’OMT-catalyzed formation of (*S*)-coclaurine, and then CNMT-catalyzed methylation on nitrogen to generate (*S*)-N-methylcoclaurine. Finally, NMCH (N-methylcoclaurine n-methyltransferase) and 4’OMT catalyze the hydroxylation and methylation of the 4′position to form reticuline [18]. Reticuline is usually used as a marker for evaluating the efficiency of an IQA biosynthesis pathway, which begins with dopamine and 4-HPAA (4-hydroxyphenylacetaldehyde). Recently, specific microbial synthesis pathways have been successfully developed for the synthesis of different IQAs, including thebaine, magnofluorine, and morphine [19].

The biosynthesis pathway of sinomenine remains unclear. Interestingly, farmers’ and pharmacists’ experience strongly indicates that bioactive ingredients differ among *S. acutum* plants with different genders. They reached a consensus that male *S. acutum* plants surpass female and undifferentiated ones as medicinal materials even in the clones obtained via cuttings. This raises the hypothesis that the regulation of sinomenine biosynthesis is gender-dependent and inspires us to the potential of using these materials to explore the regulatory mechanism of sinomenine biosynthesis.

In fact, the different sexes of dioecious individuals often exhibit varying chemical compositions and economic values [20]. For example, the flavonoid content in the leaves of female poplar is higher than that of male ones, while the levels of alkaloids in female but not male leaves are significantly reduced under severe drought stress [21]. Similarly, the flavonoid content in female plants is higher than that in male plants during different developmental stages in *Broussonetia papyrifera* [22]. Additionally, various taxoid compounds show significantly higher accumulation in female plants than male in *Taxus media* [23]. This evidence suggests that although under the same genetic background and environmental conditions, female and male plants may differ in metabolite biosynthesis, which could be governed by phytohormones, nutrition transportation, and reproductive signals.

Phytohormones play an indispensable role in regulating plant growth and yield [24]. The regulation of secondary metabolite content by phytohormones has garnered increasing interest. Numerous studies have demonstrated the important role of phytohormones in regulating secondary metabolites across diverse species [25,26,27]. For instance, ABA has a positive function in regulating the biosynthesis of monoterpene indole alkaloids in *Catharanthus roseus* [28]. Additionally, the exogenous application of ethephon (ethylene substitute) promoted the accumulation of strictosidine, catharanthine, and inblastine in *Catharanthus roseus* and upregulated the expression of key enzyme-encoding genes (*str and CrPRX*) involved in the vinblastine synthesis pathway [29]. Furthermore, auxin stimulates the production of large amounts of quinoline alkaloids in *Cinchona calisaya* Wedd [30]. Therefore, certain phytohormones might also play a role in the biosynthesis pathway of sinomenine. Therefore, we also explored the relationship between sinomenine content and phytohormone levels here.

## 2. Results

### 2.1. Morphological Characteristics of S. acutum Plants

**Figure 1:**
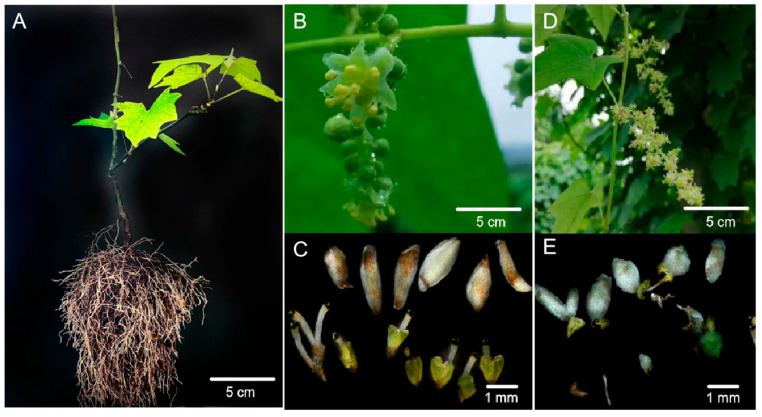
Morphology and floral microstructure of *Sinomenium acutum* plants with different genders. (**A**) An undifferentiated plant; (**B**,**C**) male flower and microanatomy; (**D**,**E**) female flower and microanatomy.

*S. acutum* is a dioecious plant. The undifferentiated plants usually start to flower after three or four years of juvenile period, during which they do not show gender characteristics or significant differences with mature plants in leaf appearance (Figure 1A). Once blooming, the *S. acutum* plants with different genders can be clearly distinguished according to the morphological characteristics of flowers. Generally, the *S. acutum* flowers are yellow in color, around 13 cm in length, and possess a panicle structure. Differently, each male flower possesses six sepals (approximately 2 mm in length) and eight to twelve stamens (approximately 1.6 mm in length) (Figure 1B,C, Table 1), while there are nine degenerated stamens and three carpels in each female flower (Figure 1D,E, Table 1). In addition, the male flowers bloom in mid-April while the females bloom in late March.

### 2.2. Overview of S. acutum Metabolome

**Figure 2:**
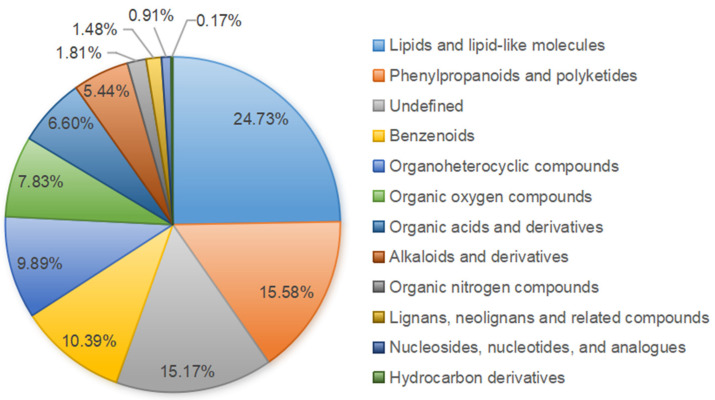
Classification and proportion of metabolites in *S. acutum*.

A total of 1,213 metabolites have been detected from the leaves and roots of *S. acutum* plants with different genders. These metabolites are classified into twelve categories (Figure 2). Among these, the category containing the most types of metabolites is lipids (24.73%), followed by phenylpropanoids and polyketones (15.58%), benzene type hydrocarbons (10.39%), organic heterocyclic compounds (9.89%), organic oxides (7.83%), organic acids and their derivatives (6.60%), and alkaloids and their derivatives (5.44%). For alkaloids, quinoline, isoquinoline, protoberberine, camptothecin, and benzopyridine alkaloids have been identified.

**Figure 3:**
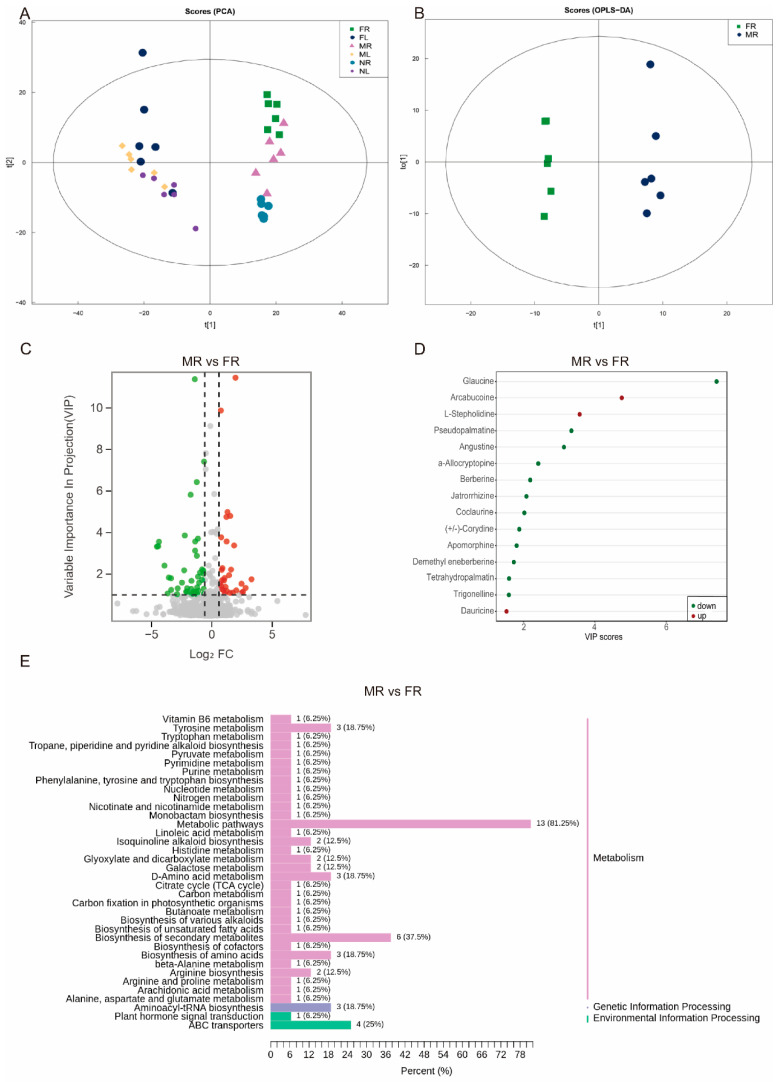
Dynamic changes of metabolome in the roots of *S. acutum*. PCA scores (**A**) and OPLS-DA (**B**) of MR vs. FR; (**C**) volcano plot for MR vs. FR. (**D**) Fifteen differential metabolites with the most significant changes for MR vs. FR. (**E**) KEGG enrichment analysis of MR vs. FR. (In LC–MS/MS profile analysis. FR, MR, and NR represent *S. acutum* male root, female root, and undifferentiated root; FL, ML, and NL represent *S. acutum* male leaf, female leaf, and undifferentiated leaf.

To reveal the differences of metabolite composition among *S. acutum* with different genders, as well as the differences between leaves and roots, principal component analyses (PCA) were conducted among the 36 collected samples. The results showed that the contribution rates of principal component 1 (t[1]) and principal component 2 (t[2]) were 39.20% and 15.44%. There is a clear distinction of metabolite composition between leaves (FL, ML, and NL) and roots (FR, MR, and NR) (Figure 3A). Furthermore, female and male plants show distinctive metabolomic profiles in the roots. On the contrary, the metabolite composition in leaves shows similar patterns across different genders. These results suggest that the differences in metabolite composition among *S. acutum* plants with different genders are concentrated in the roots. Therefore, the subsequent analysis was mainly conducted in the roots of *S. acutum* among different genders.

The multivariate statistical analysis based on the OPLS-DA model demonstrated significant metabolic differences between different genders of *S. acutum* samples in the roots (Figure 3B and Figure S1A,B). Distinct clustering patterns were observed across sample groups, with clear separation in confidence intervals and substantial inter-group dispersion. Among them, in FR vs. MR, Q2 = 0.583, R^2^X = 0.754, and R^2^Y = 0.994. In NR vs. FR, Q^2^ = 0.96, R^2^X = 0.618, and R^2^Y = 0.988. In NR vs. MR, Q^2^ = 0.462, R^2^X = 0.606, and R^2^Y = 0.888. The specific permutation plots are shown in Figure S2A–C. This indicates that the model could effectively distinguish samples from the three genders.

#### 2.2.1. Differential Accumulated Metabolites (DAMs) in *S. acutum*

Based on the OPLS-DA results, metabolites in the roots of *S. acutum* with VIP (Variable Importance in Projection) values ≥ 1 and (FC) Fold Change ≥ 1.5 or ≤0.67 were defined as differential accumulated metabolites (DAMs). Volcano plot analysis revealed distinct patterns of DAMs among the three comparison groups (Table S2): MR vs. FR: 74 DAMs (30 upregulated, 44 downregulated; Figure 3C); NR vs. FR: 97 DAMs (34 upregulated, 63 downregulated; Figure S3A); NR vs. MR: 93 DAMs (29 upregulated, 64 downregulated; Figure S3B).

Then, we focused on the alkaloid compounds from the DAMs among roots of different genders (FR, MR, NR) based on VIP values. The alkaloids including glaucine, arcabucoine, and *L*-Stepholidine were upregulated, while pseudopalmatine, angustine, and α-Allocryptopine were downregulated in the comparison of MR vs. FR (Figure 3D). Glaucine, demethyl eneberberine, and angustine were significantly upregulated, whereas *L*-Stepholidine, dauricine, and (*S*)-Scoulerine were downregulated in the comparison of NR vs. FR (Figure S3C). Upregulated alkaloids were dominated by glaucine, demethyl eneberberine, and arcabucoine, with pseudopalmatine, (*S*)-Scoulerine, and feruloyl o-methyldopamine being suppressed in the comparison of NR vs. MR (Figure S3D). Among these differential accumulated alkaloids, trigonelline, (*S*)-Scoulerine, angustine, dauricine, demecolcine, demethyl eneberberine, jatrorrhizine, tetrahydropalmatine, *L*-Stepholidine, and glaucine are common (Figure S3E,F), and approximately half of these alkaloids belong to IQAs.

#### 2.2.2. Pathway Enrichment of DAMs

To delve into the biological significance of the DAMs, we performed KEGG pathway enrichment analysis. The DAMs were mainly enriched in galactose metabolism, tyrosine metabolism, plant hormone signal transduction, and IQA biosynthesis pathways in the comparison of MR vs. FR (Figure 3E). In the comparison between NR and FR, the DAMs were significantly enriched in the TCA cycle, galactose metabolism, tyrosine metabolism, and IQA biosynthesis pathways (Figure S4A). In the comparison between NR and MR, the differential metabolites were significantly enriched in the TCA cycle, pyrimidine metabolism, and tryptophan metabolism pathways (Figure S4B). Several metabolic pathways, including galactose metabolism, tyrosine metabolism, and IQA biosynthesis, overlapped among different comparisons. This overlap may be attributed to the joint action of these pathways in forming upstream substances. In the IQA biosynthesis pathway, 3,4-dihydroxybenzaldehyde and berberine are likely key substances responsible for differences in alkaloid production.

#### 2.2.3. Prediction of Sinomenine Biosynthesis Pathway Based on Metabolome Data

**Figure 4:**
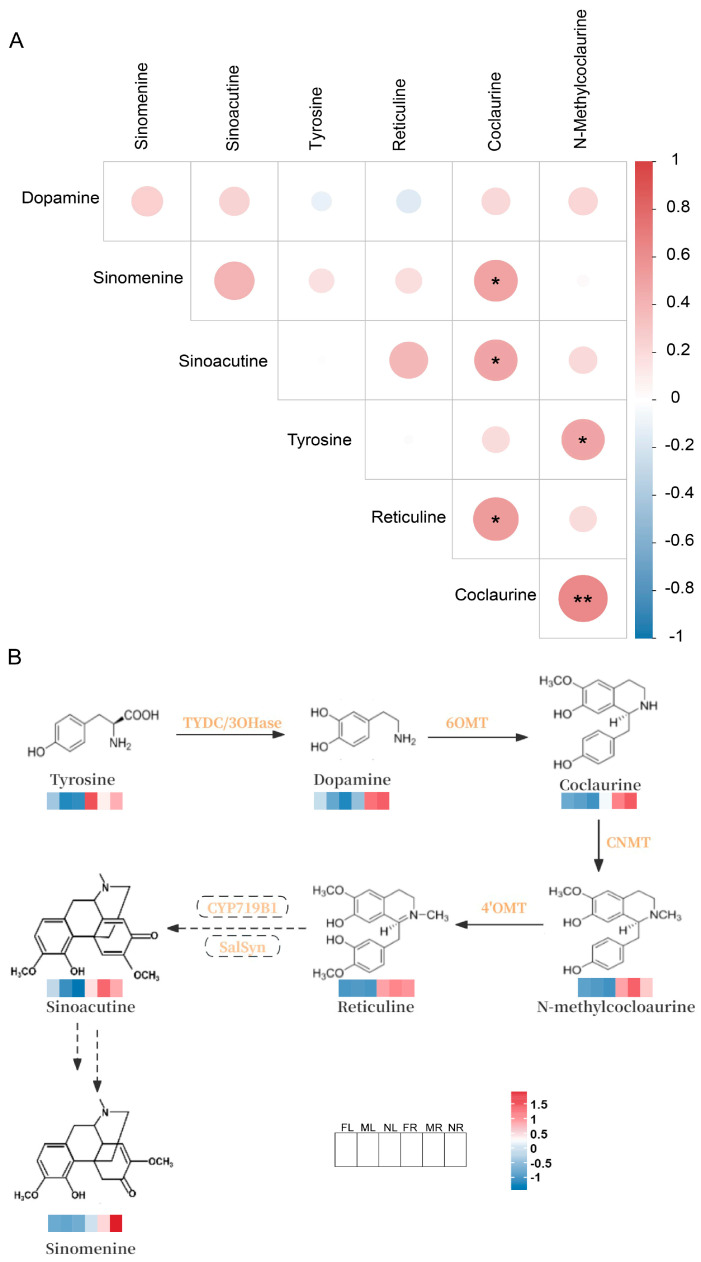
A hypothetic pathway of sinomenine biosynthesis based on correlation analysis of IQAs in *S. acutum*. (**A**) Correlation analysis of sinomenine and related metabolites; (**B**) diagram of the synthetic pathway of sinomenine and related metabolites. (In LC–MS/MS profile analysis, FR, MR, and NR represent *S. acutum* male root, female root, and undifferentiated root; FL, ML, and NL represent *S. acutum* male leaf, female leaf, and undifferentiated leaf.) The different asterisks indicate statistically significant differences with a *p* < 0.10 = *, and *p* < 0.05 = ** (one-way ANOVA, *t*-tests).

Given that sinomenine belongs to the morphinan alkaloid group within the IQA family, we focused on the IQA biosynthesis pathway based on the KEGG results. Previous studies have confirmed the common synthesis steps in the IQA biosynthesis pathway, with tyrosine serving as a precursor and undergoing a series of enzymatic reactions to produce the common intermediate, reticuline [31]. Therefore, we specifically focused on the relationship between sinomenine and other metabolites closely related to reticuline along this pathway. Five metabolites including tyrosine, dopamine, coclaurine, n-methylcoclaurine, and reticuline were analyzed. Additionally, present hypothesis suggests that sinomenine might be synthesized from sinoacutine, albeit the exact conversion process remaining unclear [32]. Thus, we analyzed the correlation between sinomenine, sinoacutine, and the aforementioned five metabolites (Figure 4A). The results showed that the strongest correlation was found between sinomenine and coclaurine, as well as the significant positive correlation between sinoacutine and coclaurine, indicating a close relationship among sinomenine and coclaurine in addition to sinoacutine.

By integrating the detected metabolites with the IQA biosynthesis pathway, the sinomenine biosynthesis pathway was hypothesized to be that sinomenine is synthesized from tyrosine as the starting substrate in the IQA pathway, with subsequent catalysis by a series of enzymes (Figure 4B).

#### 2.2.4. Correlation Analysis of IQA Content and Phytohormone Levels

**Figure 5:**
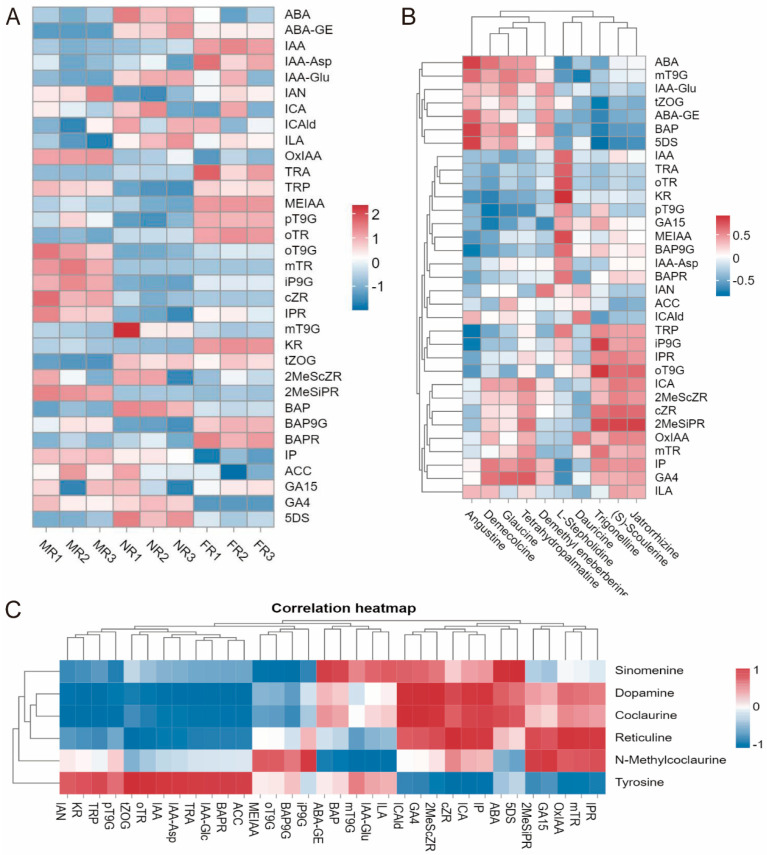
Phytohormone profiles in *S. acutum* roots and relationship between content of IQAs and phytohormone levels. (**A**) Phytohormone profiles in three genders of *S. acutum*; (**B**) correlation between phytohormone levels and content of DAMs; (**C**) correlation between phytohormone levels and content of candidate metabolites involved in the biosynthesis of sinomenine.

The phytohormone signal transduction pathway was identified in the KEGG enrichment analysis, indicating that certain phytohormones might participate in the regulation of sinomenine or other IQAs. To further explore the mechanism by which phytohormones affect sinomenine synthesis, endogenous phytohormone profiling was performed in the roots of *S. acutum* plants with different genders. A total of 34 phytohormones were detected. The results indicated significant differences of phytohormone levels among the three genders (Figure 5A).

We conducted a correlation analysis between endogenous phytohormone levels and the differential accumulated IQAs (Figure 5B). The results showed that angustine, demecolcine, glaucine, tetrahydropalmatine, and demethyleneberberine were positively correlated with ABA, mT9G (meta-Topolin-9-Glucoside), IAA-Glu (Indole-3-Acetic Acid-Glutamate), t-ZOG (trans-Zeatin-O-Glucoside), ABA-GE (Abscisic Acid Glucose Ester), BAP (6-Benzylaminopurine), and 5DS. Meanwhile, trigonelline, (S)-scoulerine, and jatrorrhizine were positively correlated with TRP (Tryptophan), iP9G (Isopentenyladenine-9-Glucoside), IPR (Isopentenyladenosine), oT9G, ICA (Indole-3-Carboxylic Acid), 2MeScZR (2-Methylthio-cis-Zeatin Riboside), cZR (cis-Zeatin Riboside), 2MeSiPR (2-Methylthio-isopentenyladenosine), OxIAA (Oxindole-3-Acetic Acid), mTR (meta-Topolin Riboside), IP (Isopentenyladenine), GA4 (Gibberellin A4), and ILA (Indole-3-Lactic Acid).

A correlation analysis was performed between the detected endogenous phytohormone levels and the content of sinomenine along with related metabolites (Figure 5C). The results indicated that sinomenine was positively correlated with ABA, 5DS, ABA-GE (Abscisic Acid Glucose Ester), BAP (6-Benzylaminopurine), ICAld (Indole-3-Carboxaldehyde), GA4, and 2MeScZR, while negatively correlated with MEIAA (Methyl Indole-3-Acetate), oT9G, BAP9G (6-Benzylaminopurine-9-Glucoside), and pT9G (para-Topolin-9-Glucoside). In addition, the metabolites closely related to sinomenine, including dopamine, coclaurine, and reticuline, were also positively correlated with the levels of identical phytohormones (ABA, 5DS, ICAld, GA4, 2MeScZR, cZR, and ICA).

#### 2.2.5. Exogenous ABA Treatment Improves Sinomenine Accumulation in *S. acutum*

**Figure 6:**
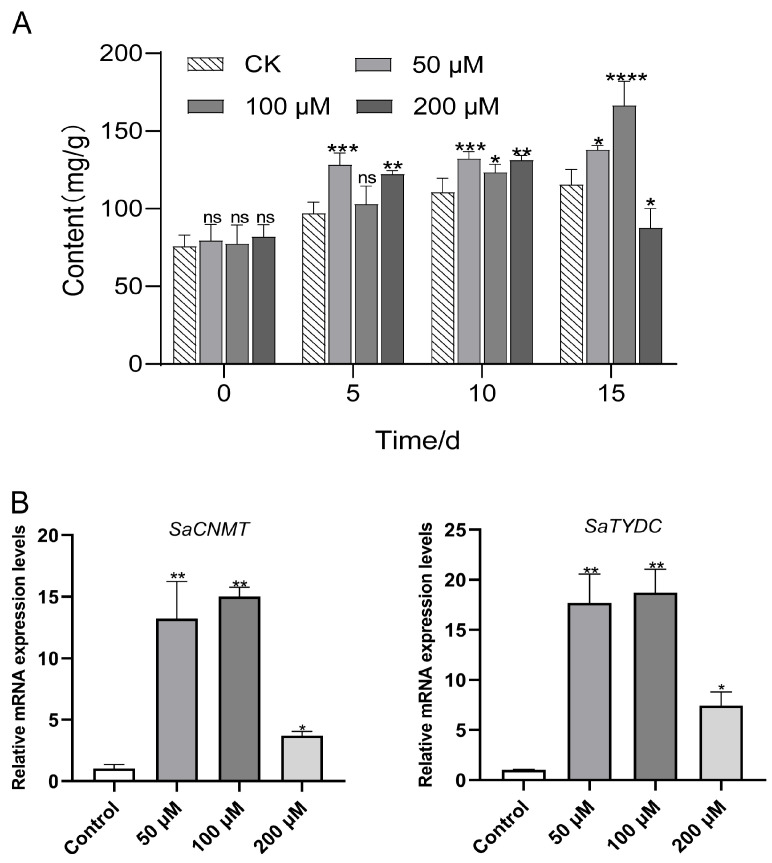
Effects of exogenous ABA treatment on sinomenine content and the expression levels of genes involved. (**A**) Effects of ABA on sinomenine content in the roots; (**B**) RT-qPCR verification of the *SaCNMT* and *SaTYDC* genes. The error bars in each column indicate the SD of two replicates. The different asterisks on the bars indicate statistically significant differences with a *p* < 0.10 = *, *p* < 0.05 = **, *p* < 0.01 = ***, *p* < 0.001 = ****, and ns as non-significant (one-way ANOVA, *t*-tests).

To further verify the function of phytohormones in sinomenine biosynthesis, exogenous ABA treatment was applied to *S. acutum* plants. A gradient concentration of ABA (50 μM, 100 μM, and 200 μM) was adopted in the treatment. The sinomenine content in both leaves and roots of *S. acutum* were improved by different degrees under ABA treatment. The sinomenine content in the roots was significantly elevated five days after treatment and continuously increased in the next ten days. The sinomenine content reached its top level (166.52 mg/g on average) after 15 d under the treatment with 100 μM ABA, which increased by 43.88% compared to the control group. However, high concentration treatment (200 μM) of ABA improved sinomenine accumulation during short-term treatment but showed an inhibition effect after 15 days (Figure 6A). In addition, we observed similar trends in sinomenine content in the leaves treated with ABA, although the content in leaves was generally lower than that in roots (Figure S5).

Then, the expression levels of identified genes involved in the sinomenine synthesis pathway were detected. The *SaCNMT* and *SaTYDC* genes encoding coclaurine N-methyltransferase and *L*-tyrosine decarboxylase gene, respectively. Following the treatment of ABA with different concentrations, the expression patterns of these genes showed consistent variation (Figure 6B). The 100 μM ABA treatment showed the most pronounced effect on the upregulation of *SaCNMT* and *SaTYDC*, whereas high concentration (200 μM) contributed less to the increase in gene expression, which is consistent with the results of sinomenine content.

## 3. Discussion

*S. acutum* is a dioecious plant. There are female, male, and undifferentiated individuals in the *S. acutum* cultivation base. Excessive harvesting driven by market demand has led to a sharp decline in the current wild *S. acutum* resources. Consequently, it is necessary to vigorously develop the artificial cultivation techniques of *S. acutum*. Production experience shows that after sex differentiation of *S. acutum* plants, there are notable differences in the content of active components, and the raw material quality of female and male plants is inconsistent, which affects the quality of subsequent processed products. The female and male plants of *S. acutum* have the same genetic background, and the morphological differences are relatively small. However, the medicinal values of plants of different genders vary greatly, which is an ideal material to study the synthesis and regulation mechanism of sinomenine, the main active substance of the plant. To reveal the key regulators determining the differences of sinomenine accumulation among *S. acutum* plants with different genders, we performed a comprehensive metabolome analysis and phytohormone profiling to analyze the changes of chemical composition and endogenous phytohormone levels during gender differentiation and to provide new insights regarding sinomenine biosynthesis regulation in *S. acutum*.

### 3.1. Metabolomics Analysis of Male and Female S. acutum Plants

There are various alkaloids in *S. acutum*, which have a wide range of effects. Sinomenine has significant therapeutic effects in the treatment of rheumatoid arthritis and other rheumatic diseases [33]. In addition, lysicamine is extracted from the climbing stems and rhizomes of *S. acutum*. Sinomenine and lysicamine both have antiproliferative activities against human colon cancer cell lines and their tumor stem cells [34]. The analysis of the distribution and changes of alkaloids in different plant tissues using metabolomics technology is helpful to clarify the metabolic patterns of key active substances. For instance, metabolomics analysis has revealed the core regulatory mechanism of steroidal sugar alkaloid metabolism in potato tubers [35]. In this study, a total of 1213 metabolites were detected in the leaves and roots of *S. acutum*, among which alkaloids accounted for 5.44%. It can be seen from the PCA results (Figure 3) that the metabolites in the leaves and roots of all materials are significantly different, proving that the accumulation of metabolites in *S. acutum* is generally tissue-specific. This result supports that the root is the main medicinal part of *S. acutum*. Notably, we found that there was no significant difference in metabolites between the leaves of plants of different genders, while there was a considerable difference in the content of metabolites in the roots. This discovery proves that the differential accumulation of key active substances in *S. acutum* is mainly concentrated in the roots; that is, the regulation of metabolite accumulation by sex differentiation mainly occurs in the roots. In the comparison of the roots between female and male plants, 74 differential compounds were identified, including many metabolites with known physiological activities. For example, the female plants had higher levels of salicylic acid, vinpocetine, and tetrahydropalmatine; the contents of substances such as salicylic acid, vinpocetine, and tetrahydropalmatine in female plants were relatively high. Salicylic acid, as a signaling molecule, can enhance the tolerance of plants to abiotic stresses such as heavy metals and high salinity [36]. This might be related to the need for stronger environmental adaptability during the reproductive stage (such as seed development and fruit formation). Vinpocetine can be used to improve neuronal function, brain inflammation, and brain oxidative stress [37]. Tetrahydropalmatine is derived from a variety of natural plants and has effects such as analgesia, anti-addiction, and anti-cancer [38]. The contents of substances such as magnoflorine, oleanolic acid, and dauricine were relatively high in male plants. Magnoflorine is a tetrahedral aporphine alkaloid that can improve Alzheimer’s disease [39]. Oleanolic acid has the effects of anti-cancer and lowering blood sugar [40], dauricine mainly has anti-inflammatory effects [41]. The high accumulation of these substances in male plants may be related to their processes such as pollen development and pollination competition, such as maintaining cell activity through anti-inflammatory and antioxidant functions. In addition, there are also differences in the above-mentioned compounds between male and female plants of sinomenine, which may also be the reason for their different medicinal values. At the same time, these identified metabolites may also serve as potential marker compounds for evaluating the efficacy of *S. acutum*. KEGG enrichment analysis was performed on the differential metabolites, and it was noted that there were two common enrichment pathways, namely, the biosynthesis pathway of IQAs and the tyrosine metabolic pathway. In previous studies on the synthesis of IQAs, it was speculated that sinomenine might have been transformed from sinoacutine [32]. Combined with the metabolomic data and previous studies, six key metabolites closely related to sinomenine synthesis were excavated, which were tyrosine, dopamine, coclaurine, N-methylcoclaurine, reticuline, and sinoacutine. Correlation analysis was conducted between six metabolites and sinomenine, and it was found that coclaurine showed the most significant positive correlation with sinomenine. Coclaurine has currently been identified as the starting metabolite in the isoquinoline biosynthesis pathway. It might be a precursor for the synthesis of sinomenine and has the same analgesic effect as sinomenine [42].

In addition, it was found that sinoacutine also has a positive correlation with sinomenine, which is consistent with the results of previous studies. Furthermore, during research on the biosynthesis of benzyl isoquinoline in *Menispermum dauricum*, a novel enzyme MdCYP80G10 was discovered that catalyzes the C2’-C4a phenolic coupling reaction of (*S*)-reticuline to generate hygrosinine. This enzyme can specifically recognize (*S*)-reticuline. Moreover, sinomenine and sinoacutine accumulate abundantly in its leaves, further verifying that sinomenine may be transformed from sinoacutine [43].

### 3.2. Analysis of Phytohormonal Differences Between the Roots of Male and Female S. acutum Plants

Phytohormones are important signal metabolites that regulate secondary metabolites in plants and play a significant role in plant growth and development, reproduction, and the accumulation of metabolites [44]. When plants differentiate between male and female, endogenous phytohormones will change and can regulate plant sex differentiation. In recent years, there have been numerous studies on the difference in the content of various phytohormones between male and female plants. For example, in the medicinal plant *Idesia polycarpa* Maxim., the content of IAA in male flowers is higher than that in female flowers, and the contents of ABA, GA3, and tZR are higher than those in male flowers [45]. The ABA content in the leaves of female *Schisandra sphenanthera* plants was significantly higher than that of male plants, while the contents of IAA, GA3, and ZR were significantly lower than those in male plants [46]. And in other plants such as *Xanthoceras sorbifolium* Bunge, IAA, ABA, and ZT are beneficial to the development of the pistil of *Xanthoceras sorbifolium* Bunge [47]. These results indicate that there are certain differences in the contents of phytohormones between dioecious plants.

In this study, the contents of IAA, MEIAA, BAPR, KR, oTR, and pT9G in female plants were higher than those in male and undifferentiated plants. On the contrary, the levels of OxIAA, oT9G, mTR, iP9G, cZR, and 2MeSiPR in male plants were higher than those in female and undifferentiated plants. Meanwhile, the results of the correlation analysis between phytohormone levels and the content of candidate metabolites showed that ABA, 5DS, and GA4 were positively correlated with the content of sinomenine. Previous studies have reported that ABA can regulate the synthesis of alkaloids. For example, exogenous ABA plays an important role in promoting the biosynthesis regulation of monoterpene indole alkaloids in *Catharanthus roseus* [28]. Spraying ABA on mature grapefruits can accelerate the accumulation of polysaccharides and polyphenols [48]. The long-term effect of ABA on alkaloid synthesis is very complex. Many physiological and biochemical processes indirectly related to alkaloid synthesis, such as photosynthesis in plants, root and stem development, and tolerance to abiotic and biotic stresses, might also be affected by ABA [49]. 5DS belongs to strigolactones (SLs), and its synthetic analogue is GR24. GR24 was used to upregulate the expression of anthocyanidin synthesis genes *Dihydroflavonol 4-Reductase* (*DFR*), *Anthocyanidin Synthase* (*ANS*), and *Transparent Testa 7* (*TT7*) to promote the synthesis and accumulation of anthocyanins in *Arabidopsis thaliana* [50]. In *Artemisia annua* leaves, GR24 treatment can improve the production of artemisinin [51]. The gibberellin treatment can significantly increase the content of total flavonoids and chlorogenic acid in both the stems and leaves in forage ramie [52]. Therefore, we speculate that ABA, 5DS, and GA4 may promote the biosynthesis of sinomenine in *S. acutum* as well.

TYDC and CNMT are two key enzymes involved in the IQA biosynthesis pathway. They play important roles in the synthesis of alkaloid active substances, mainly concentrated in medicinal plants capable of synthesizing benzyl IQAs, such as *Stephania japonica* [53] and *Opium poppy* [54]. It was found that CNMT is a key enzyme in the biosynthesis of (*S*) -reticulene in the BIA pathway [55]. In addition, transgenic apple plants overexpressing the *MdTYDC* gene showed a lower water loss rate and a higher photosynthetic pigment content to enhance the drought tolerance of apples [56]. Transcriptome sequencing of *Asarum heterotropoides* revealed that TYDC might be the key enzyme in the biosynthesis pathway of aristolochic acid. They indicate that TYDC and CNMT have multiple values in aspects such as the biosynthesis of medicinal components and plant stress resistance. Tyramine is catalyzed by TYDC and is a precursor of alkaloids such as dopamine. Its accumulation can enhance the antioxidant capacity of plants and has a synergistic effect with the stress response of ABA. The expression level of the *CNMT* gene increased in injured lotus leaves, accompanied by a significant increase in the total alkaloid content. It is speculated that CNMT also plays an important role in the alkaloid synthesis pathway [57]. We found that spraying *S. acutum* with exogenous ABA could promote the expression of *SaTYDC* and *SaCNMT* (Figure 6B). Previous studies have shown that ABA can induce the expression of many stress-resistant related genes. For example, after spraying ABA on *Nicotiana tabacum* L., the genes encoding the bHLH, bZIP, ERF, MYB, NAC, WRKY, and HSF families were all significantly upregulated [58]. It is speculated that ABA treatment further regulates the expression of key genes for sinomenine synthesis by activating these transcription factors, which plays a role in the initial step of BIA synthesis and ultimately increases sinomenine synthesis.

However, given the high complexity of plant growth and metabolic regulation, the accumulation level of specific metabolites within plants is influenced by a multi-level and multi-factor precise regulatory network [59]. In addition to the key internal factor of plant sex differentiation, factors such as genetic background and environmental factors like light, temperature, and water also play important roles [60,61]. For example, under drought stress, the contents of flavonoids (quercetin, kaempferol) and terpene insect-resistant substances (tannic acid) in the leaves of male poplar plants increased significantly, while those in female plants decreased [21]. This indicates that there are multiple factors that may lead to significant differences in the types, contents, and even spatiotemporal distribution of metabolites between plants of different genders. In this study, although our research focused on the differences related to gender differentiation, future studies can clearly control for these factors to isolate gender-specific influences.

In brief, based on comprehensive analysis of metabolomics and phytohormones, we explored the differences and possible regulatory factors of sinomenine content across female, male, and undifferentiated plants of *S. acutum*, in order to provide a theoretical basis for the rational development and utilization of *S. acutum* resources, and offer guiding significance for the cultivation, sex selection, and breeding of *S. acutum* in the future. If the targets are analgesia, anti-cancer (tetrahydropalmatine) or brain health active ingredients (vinpocetine), female plants should be given priority for planting; ff the goal is to resist Alzheimer’s disease (magnoflorine) and lower blood sugar (oleanolic acid), priority should be given to expanding male plants. Meanwhile, the proportion of female or male plants can be optimized by detecting the endogenous phytohormones in the roots of *S. acutum* to reduce the cost of eliminating ineffective plants. Therefore, in practical production, marker compounds indicated here can be used to identify the gender of undifferentiated plants. According to production needs, female or male plants can be cultivated in a targeted manner to increase the yield and extraction efficiency of the target compound and reduce resource waste. In addition, our results indicate that ABA treatment could be a potentially effective and low-cost method to increase sinomenine accumulation.

## 4. Materials and Methods

### 4.1. Plant Materials

The *S. acutum* variety Fenglong was used in this study. The three-year-old male and female plants, and one-year-old undifferentiated plants were grown at the Chenjiawan planting base in Huaihua City (27°26′ N, 109°56′ E; 307 m). We chose one-year-old *S. acutum* plants as undifferentiated because *S. acutum* seedlings differentiate into different genders after 1 year. However, the gender characteristics could be determined in three-year-old plants. Therefore, we chose one-year-old seedlings and three-year-old plants to ensure the different genders of all tested materials. The leaves and roots of *S. acutum* plants were collected from three or more individuals for each sample. Samples were collected and processed on 13 December 2022 for metabolomics. The exogenous ABA treatment on *S. acutum* was carried out in October 2024. Collected samples were mixed and immediately frozen in liquid nitrogen and stored at −80 °C for metabolites and RNA extraction. The samples were denoted as FR (roots of female plants), MR (roots of male plants), NR (roots of undifferentiated plants), FL (leaves of female plants), ML (leaves of male plants), and NL (leaves of undifferentiated plants). All the plants were grown under identical conditions as follows. The planting medium contains peat soil, perlite = 2:3, the temperature is 23 °C, the moisture content is 60–70%, and the photoperiod is 16 h light/8 h dark. All samples were treated and collected at the same time.

### 4.2. Metabolome and LC-MS Analysis

The leaves and roots were separately collocated from male, female, and undifferentiated *S. acutum* plants. Six biological replicates were set for each sample. The samples (80 mg for each) were ground into fine powder in liquid nitrogen. A total of 1000 μL methanol/acetonitrile/H_2_O (2:2:1, *v*/*v*/*v*) was added to the homogenized solution for metabolite extraction. The supernatant was dried in a vacuum centrifuge and the samples were re-dissolved in 100 μL acetonitrile/water (1:1, *v*/*v*) solvent and centrifuged at 14,000× *g* at 4 °C for 15 min to obtain the supernatant. The root and leaf samples were collected from more than five randomly selected plants for each gender. In total, 100 g and 10 g for each root and leaf sample, respectively. There were six biological replicates used for metabolomics.

Analyses were performed using an UHPLC (1290 Infinity LC, Agilent Technologies, Palo Alto, CA, USA) coupled to a quadrupole time-of-flight (AB Sciex TripleTOF 6600) in Shanghai Applied Protein Technology Co., Ltd (Shanghai, China). The samples were separated by Agilent 1290 Infinity LC ultra-performance liquid chromatography (UHPLC) on a C-18 column. The column temperature was 40 °C. The flow rate was set at 0.4 mL/min, and the injection volume was 2 μL. The mobile phase A consisted of 25 mM ammonium acetate and 0.5% formic acid in water; mobile phase B was methanol. The gradient elution procedure was as follows: 0–0.5 min, 5% B; then, B changed to 100% linearly from 0.5 to 10 min; 10–12.0 min, B was maintained at 100%; from 12.0 to 12.1 min, B changed linearly from 100% to 5%; 12.1–16 min, B was maintained at 5%. During the whole analysis, the sample was placed in an automatic sampler at 4 °C. In order to avoid the influence caused by the fluctuation of the instrument, random sequence was used for the analysis of samples. QC samples were inserted into the sample queue to monitor and evaluate the stability and reliability of the data.

The ESI source conditions were set as follows: Ion Source Gas1 as 60, Ion Source Gas2 as 60, curtain gas (CUR) as 30, source temperature: 600 °C, Ion Spray Voltage Floating (ISVF) ± 5500 V. In MS-only acquisition, the instrument was set to acquire over the *m*/*z* range 60–1000 Da, and the accumulation time for TOF MS scan was set at 0.20 s/spectra. In auto MS/MS acquisition, the instrument was set to acquire over the *m*/*z* range 25–1000 Da, and the accumulation time for product ion scan was set at 0.05 s/spectra. The product ion scan was acquired using information-dependent acquisition (IDA) with high sensitivity mode selected. The parameters were set as follows: the collision energy (CE) was fixed at 35 V with ± 15 eV; declustering potential (DP), 60 V (+) and −60 V (−); exclude isotopes within 4 Da; candidate ions to monitor per cycle: 10.

The raw MS data (wiff.scan files) were converted to MzXML files using ProteoWizard MSConvert before being imported into freely available XCMS 3.18.0 software. For peak picking, the following parameters were used: centWave *m*/*z* tolerance = 10 ppm, peakwidth = c (10, 60), prefilter = c (10, 100). For peak grouping, bw = 5, mzwid = 0.025, minfrac = 0.5 were used. CAMERA (Collection of Algorithms of Metabolite Profile Annotation) was used for the annotation of isotopes and adducts. In the extracted ion features, only the variables with more than 50% of the nonzero measurement values in at least one group were retained. Compound identification of metabolites was performed by comparing accurate *m*/*z* value (<10 ppm) and MS/MS spectra with an in-house database established using available authentic standards.

The detection of metabolites was carried out by using UHPLC-Q-TOF/MS. By comparing the experimental data with the database information through the system, including key parameters such as the retention time of metabolites, precise molecular weight (mass error < 10 ppm), secondary mass spectrometry characteristic fragments, and collision energy, the precise identification of metabolites was achieved, and the structural identification of metabolites in biological samples was completed. After normalizing the metabolomics data using Sum Normalization, we selected the VIP value ≥ 1 and FC ≥ 1.5 or ≤0.67 to screen for differential metabolites. The Kyoto Encyclopedia of Genes and Genomes (KEGG) database was used to annotate metabolites with accumulated differences and analyze enrichment pathways under specific conditions (https://cloud.metware.cn).

### 4.3. Phytohormone Level Measurement

Each root sample was prepared from 500 mg (fresh weight) of plant tissue to measure phytohormone levels. Then, 1 mL of methanol/water/formic acid (15:4:1, *v*/*v*/*v*) containing 10 μL internal standard solution with a concentration of 100 ng/mL was added for extraction. The supernatant was collected, dissolved in 100 μL of 80% methanol/water solution, and filtered through a 0.22 μm membrane. The phytohormone levels were then measured using the ESI-HPLC-MS/MS methods. There were three biological replicates used for phytohormone detection.

### 4.4. The Impact of Exogenous Hormone Treatment on the Alkaloids in S. acutum

*S. acutum* plants with the same physiological state were selected and uniformly sprayed with an ABA solution between 10:00 and 11:00 am. Samples were collected for identification on 0, 5, 10, and 15 days after treatment. Plants sprayed with 5% anhydrous ethanol were set up as a control. Three biological replicates were set up for each treatment. There were three biological replicates used for exogenous ABA treatment.

Sinomenine extraction from the samples of *S. acutum* refers to the method described in the *Chinese Pharmacopoeia* [1]. The leaves and roots are dried at 55 °C for 5 h and then ground into powder. Then, 70% ethanol was added to the powder, and the ratio of material to liquid is 1:40. The concentration of the crude drug is approximately 1 g/mL. Sinomenine content was measured using the method described by Cao et al. [62].

### 4.5. Detecting the Expression Levels of Genes Associated with Sinomenine

qPCR was used for detecting gene expression levels. Experimental operations were carried out using the cDNA synthesis kits (Accurate Biotechnology (Hunan) Co., Ltd., Changsha, China), including total RNA extraction (AG21040), cDNA synthesis, as well as qRT-PCR reaction (AG11701) and detection. A *SaActin* gene was used as an internal reference for data normalization [63]. The processing results are expressed as 2^−△△CT^ method [64], with three repetitions for each group. Primer information is shown in Table S1.

### 4.6. Data Processing and Statistics

All experimental data are presented as the means of three to six independent biological replicates with standard deviation. Statistical significance between samples was investigated by Student’s *t*-test or Duncan’s test. Student’s *t*-test or Duncan’s test was performed using the GraphPad Prism 9.00 Software (San Diego, CA, USA).

## 5. Conclusions

In summary, this study analyzed the metabolic differences in the leaves and roots among *S. acutum* plants with different genders. A total of 1213 metabolites were identified and the differentially accumulated metabolites enriched in the IQA biosynthesis pathway were specially focused on. Coclaurine is identified as a key intermediate involved in the biosynthesis of sinomenine. Significant differences in phytohormone levels among *S. acutum* plants with different genders were observed. ABA and 5DS may play a role in sinomenine biosynthesis regulation. Sinomenine accumulation is promoted through exogenous ABA treatment in both leaves and roots. The 100 μM ABA treatment showed the optimal promotion effect on sinomenine accumulation. Collectively, our findings confirm the gender-determined discrepancy of sinomenine accumulation in *S. acutum* and provide evidence for understanding the regulatory mechanism underlying this phenomenon. Meanwhile, the results of ABA treatment also suggest a potential method to improve sinomenine biosynthesis in *S. acutum*.

## Figures and Tables

**Table 1:** Morphological differences among *S. acutum* plants with different genders.

*S. acutum* Plants	Pedicel Length (cm)	Carpels (Number)	Stamens (Number)	Sepals (Number)	Total Plants Observed (Number)
Female	8–20	3	9 (degenerated)	-	183
Male	13–20	-	8–12	6	424
Undifferentiated	-	-	-	-	102

## Data Availability

The raw data supporting the conclusions of this article will be made available by the authors upon request.

## Supplementary Materials

The following supporting information can be downloaded at https://www.mdpi.com/article/10.3390/plants14121885/s1, Figure S1: The OPLS-DA analysis diagrams of *S. acutum* in different genders; Figure S2: The permutation plot of different genders of *S. acutum*; Figure S3: Analysis of differential metabolites of *S. acutum*; Figure S4: KEGG pathway enrichment results of NR vs. FR (A) and NR vs. MR (B); Figure S5: Effects of ABA on sinomenine content in the leaves of *S. acutum*; Table S1: Primer sequences of *S. acutum*; Table S2: Differential metabolites among each group.
