# Supplementary material for: Metabolomic Insights into Sexual Multi-Morphism of Sinomenine Accumulation in Sinomenium acutum
Source: Plants (Basel). 2025 Jun 19;14(12):1885. doi: 10.3390/plants14121885 (PMC12196916; doi:10.3390/plants14121885)
Supplement: Supplementary file 1 [file plants-14-01885-s001.zip › Supplementary Material .pdf]

Supplementary Materials

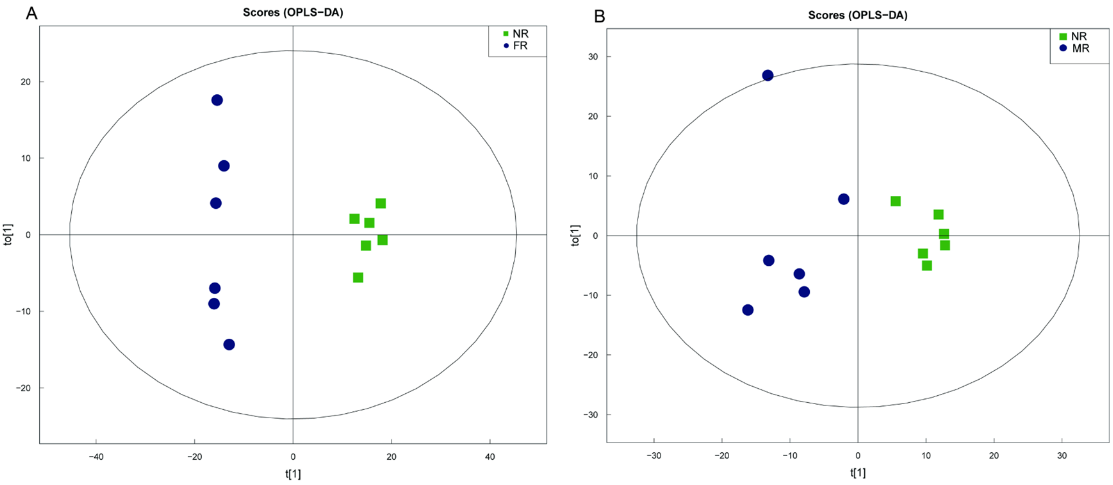

Supplementary Figure S1.

The OPLS-DA analysis diagrams of *S. acutum* among different genders.

NR vs FR**(A)** and NR vs MR **(B)**. (FR, MR and NR represent the *S. acutum* male root, female root and undifferentiated root.)

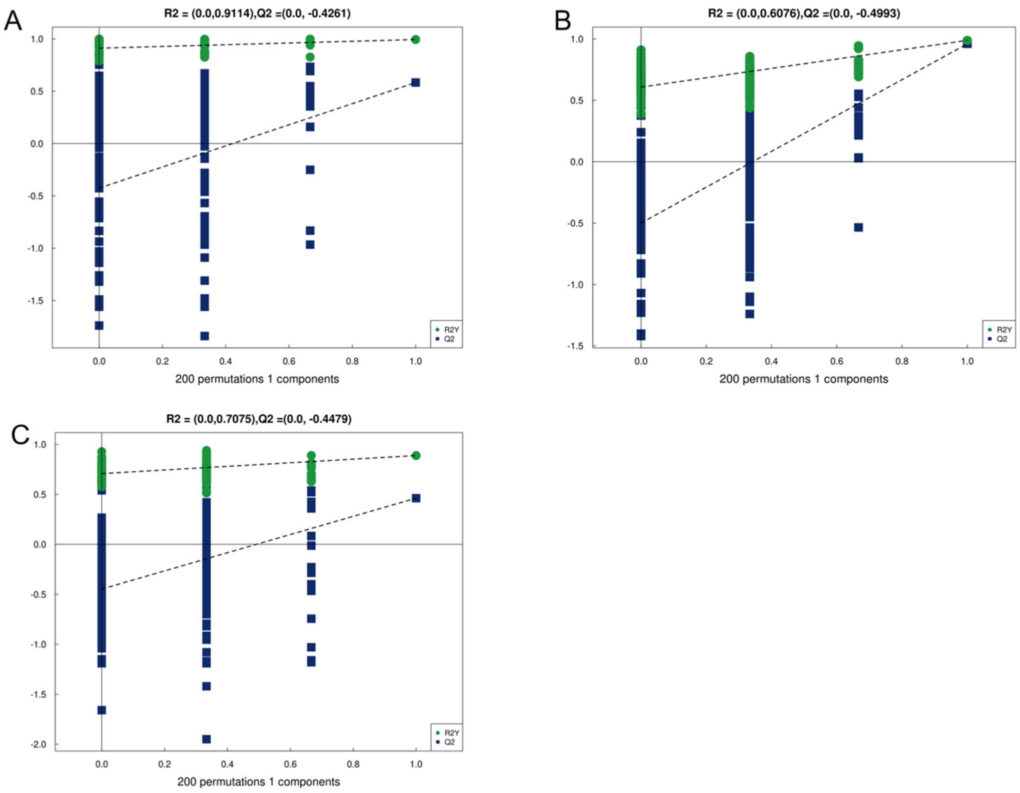

Supplementary Figure S2.

The permutation plot of the *S. acutum*.among different genders

MR vs FR**(A)**, NR vs FR**(B)** and NR vs MR **(C)**.

(FR, MR and NR represent the *S. acutum* male root, female root and undifferentiated root.).

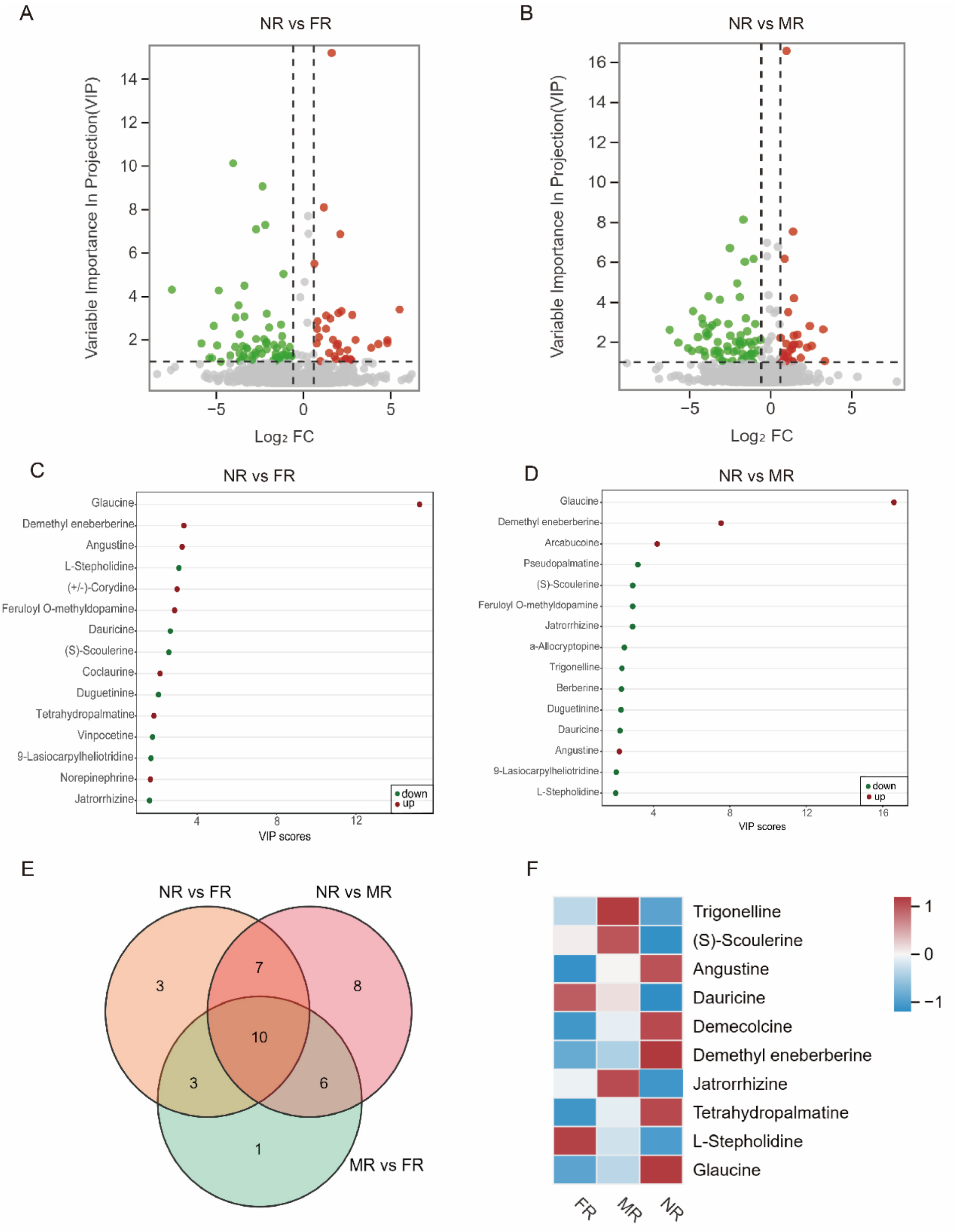

Supplementary Figure S3.

Analysis of differential metabolites of *S. acutum*. Volcano plot for NR vs FR **(A)** and NR vs MR **(B)**.; 15 differential metabolites with the most significant changes for NR vs FR **(C)** and NR vs MR **(D)**; **(E)** Venn diagram depicting the shared and the specific number of alkaloid in different comparison groups; **(F)** Heat map showing the alkaloid content in three genders. (FR, MR and NR represent the *S. acutum* male root, female root and undifferentiated root.).

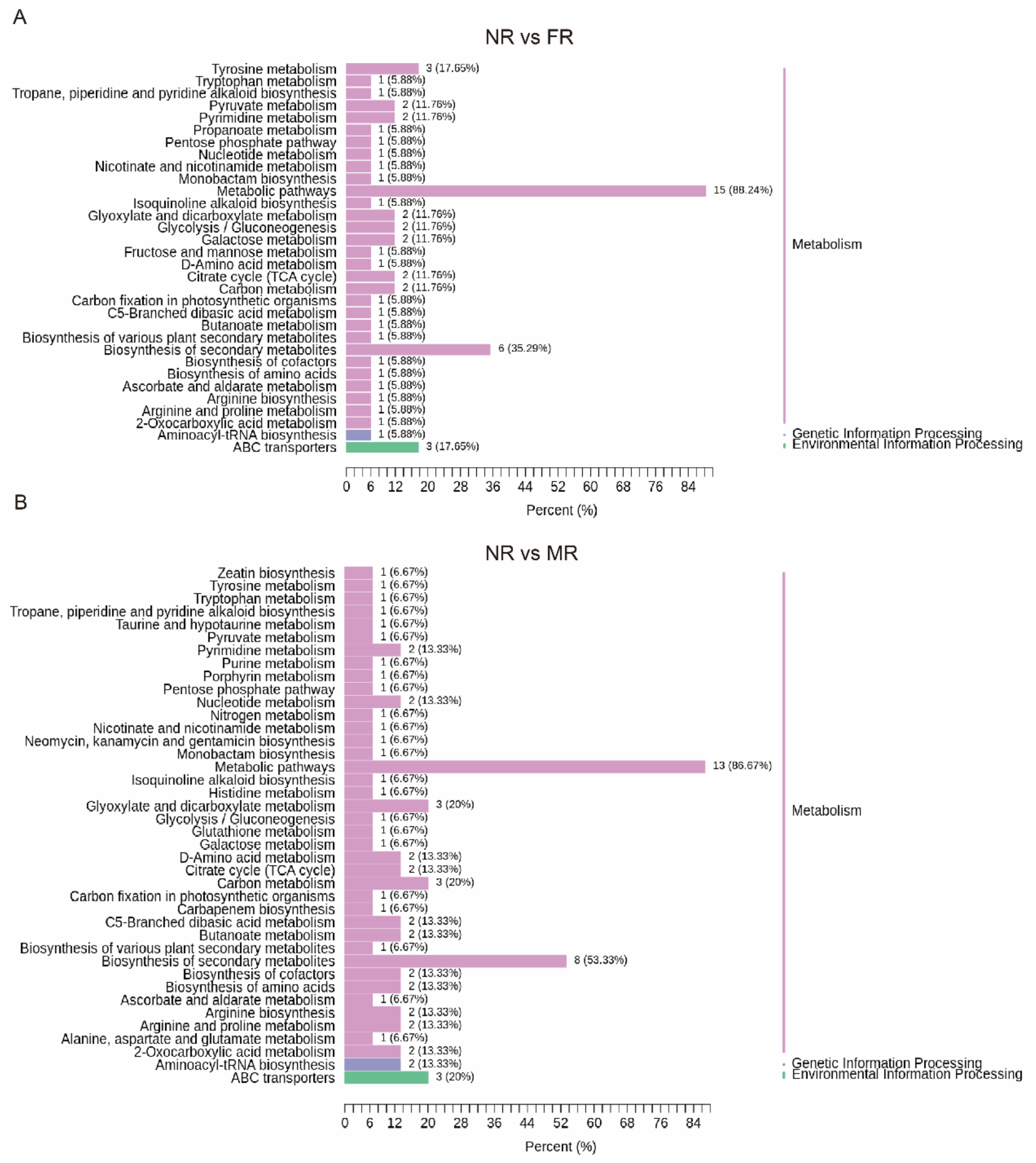

Supplementary Figure S4.

KEGG pathways enrichment results of NR vs FR **(A)** and NR vs MR **(B)**.

(FR, MR and NR represent the *S. acutum* male root, female root and undifferentiated root.).

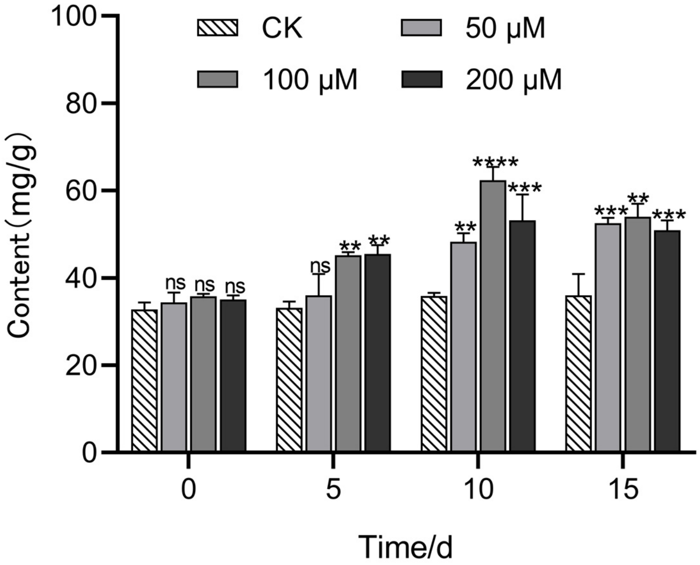

Supplementary Figure S5.

Effects of ABA on sinomenine content in the leaves of *S. acutum*.

The error bars in each column indicate SD of two replicates. The different asterisks on the bars indicate statistically significant differences with a $P < 0.10$ = *, $P < 0.05$ = ** and $P < 0.01$ = ***(one-way ANOVA, T-tests)

Supplementary Table S1. Primer sequences of *S. acutum*.

gene	primer sequence
SaActin-F	CCTCTTCCAGCCTTCCTTCAT
SaActin--R	TCTCCTTGCTCATCCTGCAG
SaTYDC- F	CTGATTCTGCACCAATCCATC
SaTYDC-R	AGCATCTTAGCAAGCCAGTCC
SaCNMT-F	AAAGCGTCTCAGATGGGGTT
SaCNMT-R	TAGTAGAGTGAGTGCTCCATAACC
